# Effect of pre-operative carbohydrate loading on aspiration risk evaluated with ultrasonography in type 2 diabetes patients: a prospective observational pilot study

**DOI:** 10.1038/s41598-022-21696-1

**Published:** 2022-10-20

**Authors:** Seohee Lee, Jin Young Sohn, Ho-jin Lee, Susie Yoon, Jae-Hyon Bahk, Bo Rim Kim

**Affiliations:** 1 Department of Anesthesiology and Pain Medicine, Seoul National University Hospital, Seoul National University College of Medicine, Seoul, Korea; 2 Department of Anesthesiology and Pain Medicine, Korea University Guro Hospital, Korea University College of Medicine, 148 Gurodong-ro, Guro-gu, Seoul, 08308 Korea

**Keywords:** Endocrinology, Medical research, Risk factors

## Abstract

Owing to concerns about delayed gastric emptying or hyperglycemia, evidence is lacking regarding whether pre-operative carbohydrate loading can be routinely administered to patients with type 2 diabetes. The objective of this study was to determine the aspiration risk and gastric volume after pre-operative carbohydrate loading in patients with type 2 diabetes. A prospective, single-center, observational cohort study. The study was conducted at a tertiary teaching hospital in Seoul, Korea, from May 2020 to May 2021. Patients (n = 49) with type 2 diabetes underwent elective noncardiac surgery. All patients were administered carbohydrate loading two hours before surgery. Once in the operating room, they underwent gastric ultrasonography to determine gastric volume. The anesthesiologists monitored the patients' glucose concentrations during and after surgery. The primary outcome was the predicted risk of aspiration. The secondary outcomes were gastric volume, antral grade, satisfaction score, and perioperative glucose profile. Forty-nine patients were analyzed. All patients had a low risk of aspiration after carbohydrate loading, as follows: 33 (67.3%) patients classified as antral grade 0 and 16 (32.7%) patients classified as antral grade 1. The median time from carbohydrate drink ingestion to ultrasound examination was 120 min (IQR 115–139). After carbohydrate loading, the median gastric volume in the right-lateral position after carbohydrate loading was 2.64 ml (IQR 0.00–32.05). The mean glucose concentrations (SD) were 134 (24) mg/dl, 159 (37) mg/dl, 150 (32) mg/dl, and 165 (36) mg/dl at baseline, after induction, 30 min after surgery, and in the post anesthesia care unit, respectively. The median satisfaction score of the patients was 5 (IQR 4–5). Pre-operative carbohydrate loading may be feasible for patients with type 2 diabetes and without complications.

Trial registration: ClinicalTrials.gov (NCT04456166). Registered on 2 July 2020.

## Introduction

Pre-operative fasting has been emphasized because of the importance of reducing the risk of complications such as aspiration in patients under general anesthesia^1^. However, previous studies have shown that prolonged fasting failed to reduce the gastric volume^2,3^. Patients with prolonged fasting experienced more thirst and hunger than did patients with short-term fasting^4^. In addition, and the catabolic state after prolonged fasting results in insulin resistance under surgical stress and contributes to increased postoperative morbidity and mortality^5^. Therefore, guidelines have recommended carbohydrate loading while highlighting a short fasting time as an alternative method to reduce hunger, insulin resistance, and discomfort^6–8^.

However, impaired gastric emptying^9^ or risk of hyperglycemia in diabetic patients are of great concern^10^. Therefore, pre-operative carbohydrate loading in diabetic patients may be problematic^11^. Although a previous study reported the safety of pre-operative carbohydrate loading in diabetic patients^12^, gastric emptying has not been examined under gastric ultrasonography (US). Gastric US is a non-invasive and reliable tool for assessing the volume of gastric contents^13,14^. The cross-sectional area (CSA) of the gastric antrum, evaluated with gastric US, could be an alternative to gastric volume^14–16^.

Therefore, we conducted an observational study to assess the risk of aspiration after carbohydrate loading in patients with type 2 diabetes mellitus by using gastric US. We hypothesized that pre-operative carbohydrate loading in diabetic patients might be tolerable in terms of potential aspiration risk and we evaluated the aspiration related findings by examining the CSA of the gastric antrum.

## Materials and methods

This study was approved by the Institutional Review Board of Seoul National University Hospital (Seoul, Korea; approval no. 2003-047-1108) on 25 May 2020 and was registered with ClinicalTrials.gov (NCT04456166). This observational study was conducted in compliance with the Declaration of Helsinki and the guidelines for Good Clinical Practice.

### Patient characteristics

Adult patients (≥ 18 years old) with type 2 diabetes taking oral hypoglycemic agents who were scheduled to undergo elective laparoscopic cholecystectomy or video-assisted thoracic surgery (VATS) in a tertiary teaching hospital (Seoul National University Hospital, Seoul, Korea) from May 2020 to May 2021 were screened for eligibility. All patients were originally scheduled for carbohydrate loading 2 h before surgery as a guideline. After obtaining written informed consent, 60 patients with ASA physical status II–III were enrolled. The exclusion criteria were outpatient surgery, pre-existing conditions associated with gastric emptying delay such as gastric esophageal reflux disease, previous esophageal or gastric surgery, functional dyspepsia, and history of pancreatic surgery or neurosurgery. Patients with anticipated difficult airways such as body mass index > 35 kg/m^2^, Mallampati class ≥ III, thyromental distance < 6.5 cm, or history of oropharyngeal surgery were also excluded. A carbohydrate-rich drink (200 ml, 12.8% carbohydrates, 50 kcal·100 ml; Nucare NoNPO^Ⓡ^; Daesang Wellife, Seoul, Korea) was provided to the patients. The investigator instructed them to drink 2 h before the scheduled time of the surgery. The duration of intake was recorded by ward nurses.

### Ultrasonography examination

**Figure 1:**
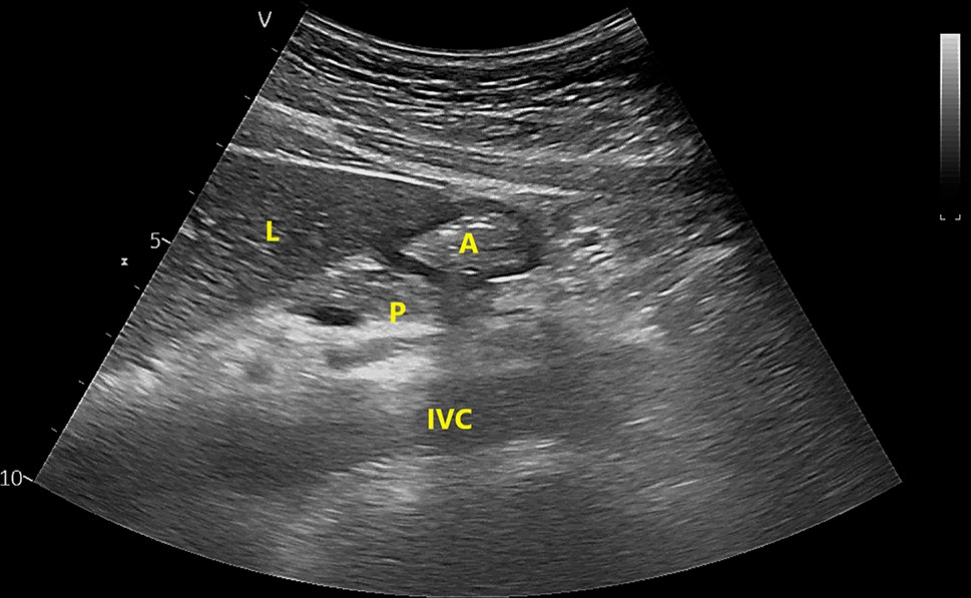
Ultrasound image of the gastric antrum in the epigastric area, obtained in the sagittal or parasagittal plane. A, antrum; L, liver; P, pancreas; IVC, inferior vena cava. The antrum is between the left lobe of the liver anteriorly and the pancreas posteriorly at the level of the aorta or the inferior vena cava.

**Figure 2:**
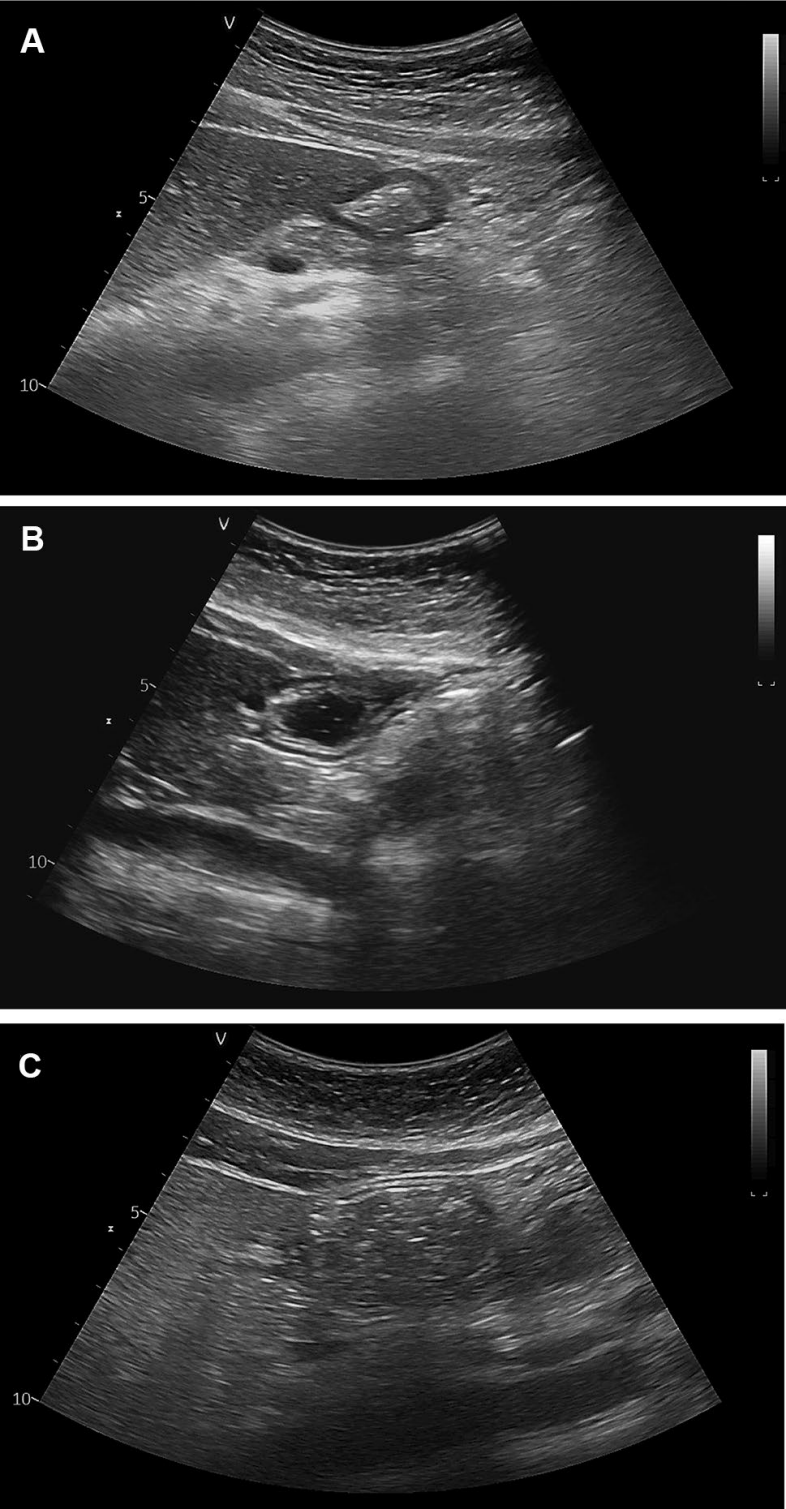
Ultrasound image of the gastric antrum in the epigastric area obtained in a sagittal or parasagittal plane according to gastric contents. The empty antrum (grade 0) is presented (**A**) in the right lateral decubitus, The antrum with minimal, insignificant amount of fluid (grade 1) appears (**B**) in the right lateral decubitus; The antrum with significant fluid content (grade 2) is detectable (**C**) in the right lateral decubitus.

Before the induction of general anesthesia, an anesthesiologist with previous experience in gastric ultrasound (S Lee) assessed a patient’s gastric volume with real-time ultrasound (Vivid T9; GE Healthcare, Wauwatosa, WI, USA) in the operating room. A convex probe (C1-5-RS Probe; GE Healthcare, Milwaukee, WI, USA) with a frequency of 1.5–5.0 MHz was placed to create a sagittal view of the epigastric region in the supine position. The antrum is usually best visualized in the sagittal or parasagittal plane between the left lobe of the liver and pancreas at the level of the aorta or inferior vena cava^15^. To acquire a true antral CSA, the patient was then positioned in the right lateral decubitus (RLD) position with the probe tilted and rotated perpendicular to the long axis of the antrum. After the examination, the antral CSA was independently measured in the obtained images by two investigators (S Yoon and BR Kim) using the traditional ‘free tracing’ tool (Fig. 1)^14^. The gastric volume was estimated, based on the average value of the measured CSA, as described in previous studies^15,16^ which report that the antral CSA and gastric volume have a linear correlation. Each patient’s antrum was then graded as 0, 1, and 2, using the method described in a previous report^17^, to assess the risk of aspiration. The grades were defined using the ‘antral grading system’, which is based on the gastric US assessment of gastric content and volume, depending on position: at grade 0, the antrum appears empty in the supine and in the right lateral decubitus positions (Fig. 2A); at grade 1, gastric fluid is visible only in the right lateral decubitus position, which suggests a small fluid volume (Fig. 2B); and at grade 2, gastric fluid in the antrum is visible in the supine and in the right lateral decubitus positions, which suggests a larger fluid volume (Fig. 2C)^17^.

### General anesthesia and glucose management protocol

Once the ultrasound examination of the gastric volume was completed, general anesthesia was induced, using a predetermined protocol. All patients received the standard perioperative care, which included routine monitoring for surgery such as the bispectral index (Coviden, Mansfield, MA, USA), electrocardiography, pulse oximetry, temperature probe, and end-tidal carbon dioxide monitoring. Arterial catheterization was administered selectively, depending on the type of surgery or patient comorbidities. After preoxygenation with 100% oxygen, then propofol (1.5–2.0 mg/kg), fentanyl (1.0–2.0 μg/kg), or target-controlled infusion of remifentanil (i.e. the Minto model, effect-site concentration up to 4.0 ng/ml) was administered intravenously. After confirming the loss of consciousness, rocuronium (0.6–0.8 mg/kg) was administered. Rapid sequence induction was planned for patients at a high risk of aspiration, defined as grade 2, based on the ultrasonography grading system. Palonosetron 0.075 mg (Palseron; Samyang Holdings, Seoul, Korea) and dexamethasone (5 mg; Daewon Pharmaceutical Co., Inc., Seoul, Korea) were used to prevent postoperative nausea and vomiting (PONV).

The patients were intubated and ventilated with 50% oxygen and air. Patients were mechanically ventilated at a tidal volume of 6–8 ml/kg and a respiratory rate of 10–20 breaths min^-1^ with an I:E ratio of 1:2 to titrate the end-tidal carbon dioxide pressure to 35–45 mmHg. Anesthesia was maintained with an intermittent fentanyl bolus or target-controlled infusion of remifentanil and sevoflurane. The bispectral index values were maintained between 40 and 60. At the end of surgery, patients received sugammadex (2.0 mg/kg) for the reversal of the neuromuscular blockade.

**Figure 3:**
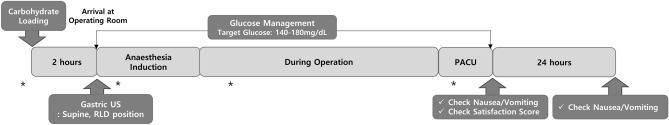
Schematic diagram of the study protocol. RLD, right lateral decubitus; US, ultrasonography; PACU, post anesthesia care unit.; IU, international unit. *Asterisks represent the time points of blood samples for glucose measurement.

The preoperative glucose concentration was regulated, using the predetermined protocol of our institution, with consideration of the type of surgery and whether the patient’s diabetes was well controlled. Among patients undergoing VATS, 12 patients with high glucose concentrations were administered the Alberti regimen combining glucose, insulin, and potassium. Oral hypoglycemic agents were maintained until 1 day before the surgery, with blood glucose concentrations monitored every 2–3 h during fasting. The blood glucose concentration was measured at the following time points: before carbohydrate loading (i.e. pre-operative), 1 min after tracheal intubation (i.e. 2 h after carbohydrate loading), 30 min after the incision (i.e. intraoperative), and in the post-anesthesia care unit (i.e. postoperative). A blood sugar test kit (Accu-Chek Inform II Meter; Roche Diagnostics, Mannheim, Germany) or a point-of-care blood gas analyzer (Gem® Premier™ 3000; Instrumentation Laboratory, Bedford, MA, USA) was used for the blood glucose measurements. To achieve a glucose concentration target of 140–180 mg/dl^18,19^, regular insulin was administered intravenously during the intraoperative and postoperative periods, following a predetermined protocol (Fig. 3).

### Study outcomes

The primary outcome was predicted risk of aspiration based on calculated gastric volumes and grade, as assessed using gastric US before the induction of general anesthesia. The aspiration risk was classified as follows: low risk was grade 0 or 1 and an estimated total gastric fluid volume ≤ 1.5 ml/kg, and high risk was grade 2 or an estimated total gastric fluid volume > 1.5 ml/kg^17^. The secondary outcome was the gastric volume, which was calculated by the linear model reported in a previous article (gastric volume (ml) = 27.0 + 14.6 × (right lateral) CSA − 1.28 × age)^15^, using the average value of two antral CSAs measured by two investigators independently. In addition, data were collected on age, sex, height, weight, body mass index, comorbidities, pre-operative condition, and diabetic profiles including duration of diabetes, hemoglobin A1c, and diabetes-related complications. Perioperative serum glucose concentration, perioperative insulin use, and patient satisfaction score regarding pre-operative carbohydrate loading by using a 6-point numeric rating scale (0 = totally dissatisfied, 5 = totally satisfied) were also documented. The incidence and severity of PONV and aspiration were investigated during the first 24 h postoperatively.

### Statistical analysis

Continuous data are presented as the mean and the standard deviation (SD), or as the median and interquartile range (IQR) after confirming the normality assumption by using the Shapiro–Wilk test. Categorical data were expressed as percentages and counts.

A previous study showed the performance of gastric antral area measurement using ultrasonography to predict the gastric fluid volumes by performing linear regression curves^20^. According to the study, the area under the receiver operating characteristics (ROC) curve was 0.86 (95% CI 0.74–0.94) to detect the gastric volume of > 1.5 ml/kg in parturients at RLD position. In this present study, we assumed the area under the ROC curve as 0.74, the lowest limit value from the aforementioned study, to predict the high risk of aspiration as we followed different estimation method^15^^,^^17^ With a significance of 0.05 and a power of 0.9, 54 patients were calculated to be required. Considering drop-out rate of 10%, we planned to collect data from 60 patients.

All statistical analyses were conducted using the MedCalc® version 20.026 (Mariakerke, Belgium) and SPSS software (ver. 25.0; IBM Corp., Armonk, NY, USA).

### Ethics approval and consent to participate

This prospective observational study was approved by Institutional Review Board of Seoul National University Hospital (Seoul, Korea; approval no. 2003-047-1108) on 25 May 2020.

## Results

**Figure 4:**
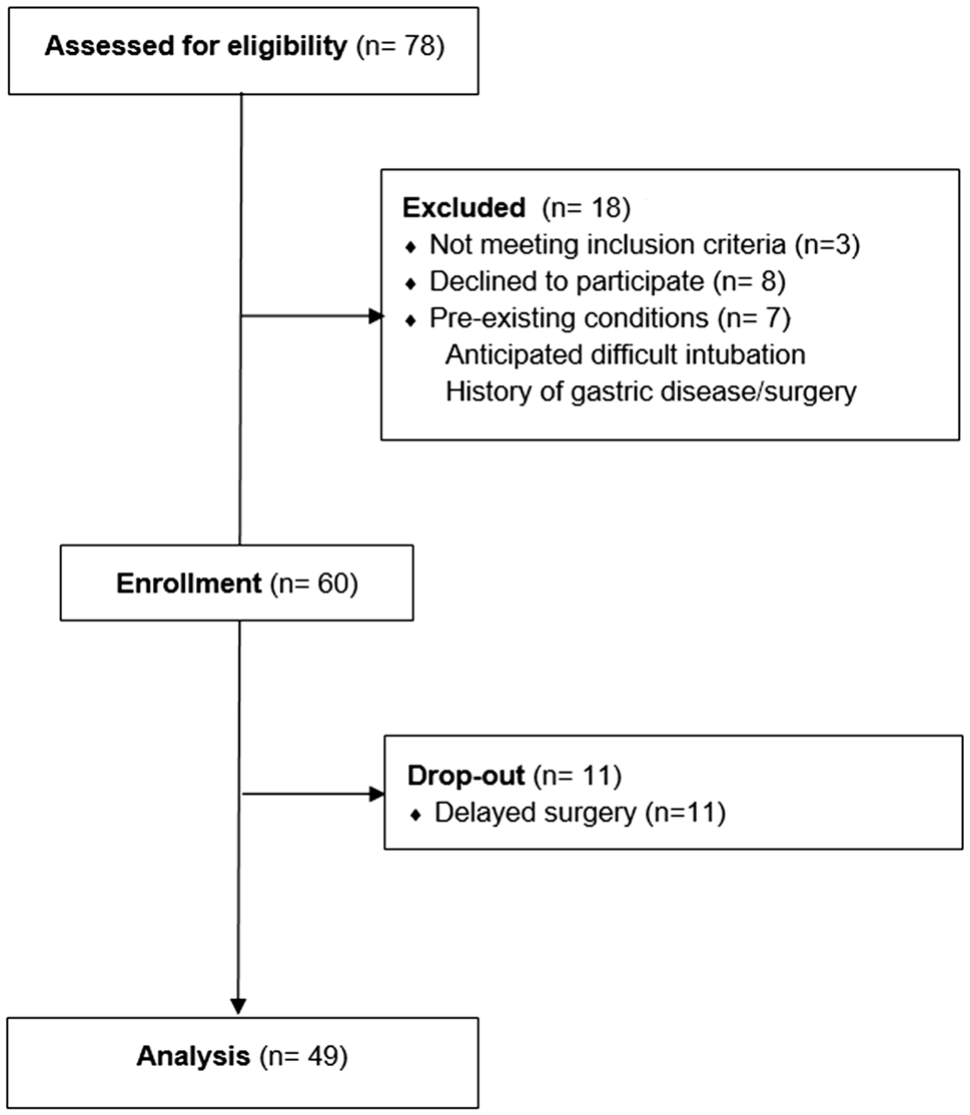
The Consolidated Standards of Reporting Trials (CONSORT) diagram of enrolment.

Seventy-eight patients were identified, of whom 60 patients were enrolled. Eleven patients dropped out because of delayed surgery (*n* = 11) (Fig. 4). Therefore, 49 patients were included in the analysis.

The surgical procedures consisted of 44 (89.8%) VATS and 5 (10.2%) laparoscopic cholecystectomies. The patients’ demographics and baseline characteristics, based on the grading system, are presented in Table 1. The average (SD) duration of diabetes in our patients was 11 (7) years and diabetes-related complications were diagnosed in 18 (36.7%) patients. The median time from carbohydrate drink ingestion to ultrasound examination was 120 (IQR 115–139) min (Table 2). All patients were found to have calculated gastric volumes that are consistent with a low predicted risk of aspiration (i.e. grade 0 and grade 1) in the ultrasound assessment. Of the 49 patients, 33 (67.3%) patients were classified as grade 0 and 16 (32.7%) patients were classified as grade 1. No patient was classified as grade 2 (Table 2). The mean (SD) CSA values in the supine and right lateral position were 3.96 (1.54) cm^2^ and 4.63 (2.06) cm^2^, respectively (Table 2). The median value of the estimated gastric volume after carbohydrate loading was 2.64 (IQR 0.00–32.05) ml.

**Table 1:** Patient demographics and baseline characteristics.

	All (*n* = 49)	Grade 0* (*n* = 33)	Grade 1* (*n* = 16)
Age, years	68 (8)	68 (9)	69 (7)
Female	17 (34.7%)	13 (39.4%)	4 (25.0%)
Height, cm	162.8 (8.4)	162.1 (8.3)	164.2 (8.5)
Weight, kg	67.3 (10.8)	68.0 (10.5)	65.9 (11.5)
BMI, kg/m^2^	25.4 (3.7)	25.9 (3.7)	24.4 (3.6)
**Comorbidities**			
Hypertension	32 (65.3%)	25 (75.8%)	7 (43.8%)
Hyperlipidemia	14 (28.6%)	12 (36.4%)	2 (12.5%)
Coronary artery disease	6 (12.2%)	3 (9.1%)	3 (18.8%)
Cerebrovascular disease	3 (6.1%)	1 (3.0%)	2 (12.5%)
**Preoperative condition**			
Mild dyspepsia	3 (6.1%)	3 (9.1%)	0 (0%)
Preoperative nausea**	0 (0%)	0 (0%)	0 (0%)
**Diabetic profiles**			
Duration of diabetes, years^†^	11 (7)	10 (6)	11 (10)
Preoperative HbA1c, %	7.1 (0.9)	7.2 (1.0)	6.9 (0.7)
Diabetes-related complications^‡^			
Nephropathy	8 (16.3%)	5 (15.2%)	3 (18.8%)
Retinopathy	3 (6.1%)	1 (3.0%)	2 (12.5%)
Neuropathy	11 (22.4%)	9 (27.3%)	2 (12.5%)
Renal function tests			
Preoperative creatinine, mg/dl	0.89 (0.24)	0.86 (0.23)	0.95 (0.24)
Preoperative eGFR, ml/min/1.73 m^2^	81.4 (16.1)	83.7 (16.5)	76.5 (14.4)

Data are presented as mean (SD), or number (%). BMI, body mass index; eGFR, estimated glomerular filtration rate; HbA1c, Hemoglobin A1c.

*The grades were defined as ‘antral grading system’ based on ultrasound assessment of gastric content and volume depending on position; grade 0—the antrum appeared empty on both supine and right lateral decubitus positions; grade 1—gastric fluid was visible on the right lateral decubitus position only, suggesting a small fluid volume.

**Two missing values in the grade 0 patients.

^†^Four missing values in the grade 0 patients.

^‡^Defined as patients who have been diagnosed with diabetes-related complications.

**Table 2:** The outcome assessment after carbohydrate loading in diabetic patients before general anesthesia.

	All (*n* = 49)	Grade 0 (*n* = 33)	Grade 1 (*n* = 16)
**Risk evaluation**			
Aspiration risk (Low/High)*	(49/0)	(33/0)	(16/0)
Grade (0/1/2)**	(33/16/0)		
**Gastric volume**			
Antral CSA, Supine (cm^2^)	3.96 (1.54)	3.40 (1.30)	5.12 (1.38)
Antral CSA, Right lateral (cm^2^)	4.63 (2.06)	3.68 (1.34)	6.55 (1.94)
Predicted gastric volume (ml)	2.64 (0.00–32.05)	0.00 (0.00–7.62)	40.27 (8.99–55.52)
**Time variables**			
Time interval^†^ (min)	120 (115–139)	120 (115–140)	120 (113–138)
**Glucose concentration (mg/dl)**			
Preoperative	134 (24)	137 (27)	130 (18)
After induction^‡^	159 (37)	162 (37)	153 (38)
Intraoperative	150 (32)	154 (32)	142 (31)
Postoperative	165 (36)	170 (40)	156 (21)
**Perioperative peak glucose level (mg/dl)**	167 (155–198)	172 (157–202)	163 (147–185)
**Insulin administration (IU)**			
Perioperative insulin use	18 (36.7%)	13 (39.4%)	5 (31.3%)
**Postoperative outcomes**			
Nausea	8 (16.3%)	6 (18.2%)	2 (12.5%)
Vomiting	0 (0%)	0 (0%)	0 (0%)
Satisfaction score	5 (4–5)	5 (4–5)	5 (4–5)

Data are presented as mean (SD), median (IQR), or number (%).

CSA, cross-sectional area; PACU, post anesthesia care unit; IU, International Unit.

*The aspiration risk was classified as follows: Low risk—grade 0 or 1, or estimated total gastric fluid volume was ≤ 1.5 ml/kg; High risk—grade 2, or estimated total gastric fluid volume was > 1.5 ml/kg.

**The grades were defined as ‘antral grading system’ based on ultrasound assessment of gastric content and volume depending on position; grade 0—the antrum appeared empty on both supine and right lateral decubitus positions; grade 1—gastric fluid was visible on the right lateral decubitus position only, suggesting a small fluid volume.

^†^Time interval was defined as the duration between carbohydrate loading time and the time of assessment of antrum.

^‡^One missing value in the grade 0 patients.

The blood glucose concentrations at each time point are shown in Table 2. The median peak glucose concentration in the perioperative period was 167 (IQR 155–198) mg/dl. Insulin was administered at least once in 36.7% (18/49) of patients during the perioperative period (Table 2). Most patients who required insulin administration required only 1 IU and were injected up to 5 IU. No patient experienced postoperative vomiting; however, 8 (16.3%) patients had postoperative nausea. No cases of regurgitation or aspiration of gastric contents occurred (Table 2). The median satisfaction score of the patients was 5 (IQR 4–5) (Table 2).

## Discussion

In this study, we evaluated gastric content and volume by using bedside gastric US to assess the risk of aspiration after carbohydrate loading in patients with well-controlled type 2 diabetes. The current study demonstrated that pre-operative carbohydrate loading did not increase the risk of aspiration. Furthermore, the patients satisfied with the pre-operative carbohydrate drink had a median satisfaction score of 5. Therefore, carbohydrate loading may be a good option and a safe strategy to alleviate discomfort from pre-operative fasting in patients with type 2 diabetes.

Several reports reveal that pre-operative carbohydrate loading does not increase gastric residual volume, cause adverse events, or delay gastric emptying in noncardiac and cardiac surgeries^21,22^. Furthermore, pre-operative carbohydrate loading instead reduces the gastric volume in nondiabetic patients^23^ and in pediatric patients^24^ or term parturients^25^. Nevertheless, the guidelines regarding carbohydrate drinks 2 h before surgery have been applied to a limited extent in patients with diabetes because of concerns of delayed gastric emptying^5,26^. Investigators in a previous study^12^ reported that patients with diabetes do not have delayed gastric emptying after pre-operative carbohydrate loading, compared with healthy people. However, using an indirect variable to evaluate gastric emptying and intestinal paracetamol absorption has a limitation in patient characteristics such as metabolism rate or digestive disorder of the intestine. Therefore, our study has its implication in that it appears to be the first study to directly evaluate gastric contents and volume under sonography, which is a useful non-invasive tool to evaluate perioperative aspiration risk^14^. In addition, patients included in this study had diabetes for an average of more than 10 years, and 22.4% of patients had been diagnosed with diabetic neuropathy which is known to be closely associated with diabetic gastroparesis^27^. Our findings reassure that preoperative carbohydrate loading did not increase the gastric volume to a degree that increased the risk of aspiration.

The advantages of pre-operative carbohydrate loading have been well studied. Insulin resistance resulting in poor glucose control is associated with complications^28^, and pre-operative carbohydrate loading decreases pyruvate dehydrogenase expression related to the citric acid cycle, thereby improving improved insulin sensitivity^29,30^. A previous study^31^ reported that pre-operative carbohydrate loading in patients undergoing gastrectomy attenuated postoperative insulin resistance, particularly in patients who were originally insulin-resistant. In addition, pre-operative carbohydrate loading may not increase the incidence of hyperglycemia, compared with fasting. In our study, the mean (SD) blood glucose concentrations before pre-operative carbohydrate intake and after induction were 134 (24) mg/dl and 159 (37) mg/dl, respectively, which were similar to the findings of a previous study that reported 144 (56) mg/dl and 151 (56) mg/dl, respectively^32^. With regard to the incidence of hyperglycemia, defined as a serum glucose concentration > 200 mg/dl^33^, the aforementioned article reported hyperglycemia in 17.4% of patients who had carbohydrate loading 2 h before surgery and in 16.7% of patients who fasted pre-operatively^32^. The incidence of hyperglycemia in the current study was similarly 12.2% (6/49 patients) and only one-third of all patients received insulin care as a predetermined protocol.

Pre-operative carbohydrate loading showed an advantage in the quality of perioperative management such as PONV or satisfaction score after surgery. PONV is a common complication after general anesthesia, and has been reported in up to 30% of patients undergoing surgery^34–36^. Previous studies have shown that, compared with a placebo, pre-operative carbohydrate consumption improved well-being and reduced PONV in patients undergoing laparoscopic cholecystectomy^37,38^. However, the incidence of PONV during the first 24 h postoperatively was 16.3% (8/49 patients), which was similar to the incidence in a previous study conducted in our institution^39^. A limitation of this observational study was that risk factors for PONV could not be controlled; therefore, a causative relationship was inconclusive. Clinicians should be cautious in overinterpreting when applying this result to actual clinical settings. In addition, several randomized controlled trials have proven that pre-operative carbohydrate loading reduced thirst, hunger, and feelings of weakness with high satisfaction^40,41^. Most patients were similarly satisfied with carbohydrate drinks with a median postoperative satisfaction score of 5 (IQR 4–5) in this study.

Our study has several limitations. First, it is an observational study with a limited sample size; therefore, providing carbohydrate drinks to all diabetic patients before surgery is difficult to strongly recommend. Nevertheless, this study provides evidence that pre-operative carbohydrate drinks can be administered to patients with well-controlled type 2 diabetes within the current fasting guidelines. Second, only a single anesthesiologist conducted all gastric US examinations; therefore, the results may be affected by operator dependency. In our study, gastric US was conducted by one anesthesiologist, followed by confirmation by other anesthesiologists to compensate for bias of the researchers. In addition, the reliability of gastric volume consecutive US assessment by clinical anesthesiologists has already been proven^42^. Third, there was no gastric ultrasonography to determine gastric volume before the carbohydrate loading. Fourth, patients were injected with dexamethasone to prevent postoperative nausea. This practice may affect the serum glucose concentration or the incidence of PONV in patients with diabetes. However, the effects of dexamethasone on glucose concentrations in patients with diabetes are minimal^43^, and fewer cases of hyperglycemia requiring insulin treatment occurred in this study. Fifth, all patients enrolled in the study were only patients with type 2 diabetes, even though diabetic gastroparesis is more often observed in patients with type 1 diabetes than in patients with type 2 diabetes^44^. Therefore, further study may be needed to confirm the same results for patients with type 1 diabetes. Sixth, our study only investigated the perioperative serum glucose concentration, but did not assess the parameters related to insulin resistance such as normoglycemic hyperinsulinemia two-step clamp methods^45^. Therefore, the current study has limitations in presenting evidence of glucose control, based on the reduction of insulin resistance under carbohydrate loading. Lastly, 200 ml of carbohydrate drinks were administered without considering the patient's weight; therefore, the findings of this study should be carefully interpreted because the incidence of hyperglycemia could be underestimated.

In conclusion, we provided a prospective sonographic assessment of gastric volume and evaluated the risk of aspiration in patients with well-controlled type 2 diabetes after pre-operative carbohydrate loading. Pre-operative carbohydrate loading did not increase gastric volume in patients with type 2 diabetes. This strategy may be feasible to enhance patient satisfaction without increasing the risk of pulmonary aspiration and hyperglycemia during general anesthesia.

## Data Availability

The datasets used and analyzed during the current study are available from the corresponding author on reasonable request.

## Abbreviations

CSA: Cross-sectional area
IQR: Interquartile
PONV: Postoperative nausea and vomiting
SD: Standard deviation
RLD: Right lateral decubitus
ROC: Range receiver operating characteristics
US: Ultrasonography
VATS: Video-assisted thoracic surgery
